# The Relationship Between ADAMTS13 Activity and Overall Cerebral Small Vessel Disease Burden: A Cross-Sectional Study Based on CSVD

**DOI:** 10.3389/fnagi.2021.738359

**Published:** 2021-10-08

**Authors:** Wenbo Sun, Yufan Luo, Shufan Zhang, Wenmei Lu, Luqiong Liu, Xiaoli Yang, Danhong Wu

**Affiliations:** ^1^Department of Neurology, Shanghai Fifth People’s Hospital, Fudan University, Shanghai, China; ^2^Department of General Medicine, Shanghai Fifth People’s Hospital, Fudan University, Shanghai, China

**Keywords:** cerebral small vessel disease, ADAMTS13, von Willebrand factor, overall cerebral small vessel disease burden, white matter hyperintensity (WMH), subcortical infarction

## Abstract

**Objectives**: This study aimed to investigate the association between plasma von Willebrand factor (VWF) level, ADAMTS13 activity, and neuroimaging features of cerebral small vessel disease (CSVD), including the CSVD neuroimaging markers and the overall CSVD burden.

**Methods**: CSVD patients admitted to our hospital from 2016 to 2020 were recruited. Plasma VWF level and ADAMTS13 activity were measured. The overall effect of CSVD on the brain was described as a validated CSVD score. We evaluated the association between VWF levels, ADAMTS13 activity, and the increasing severity of CSVD score by the logistic regression model.

**Results**: We enrolled 296 patients into this study. The mean age of the sample was 69.0 years (SD 7.0). The mean VWF level was 1.31 IU/mL, and the ADAMTS13 activity was 88.01 (SD 10.57). In multivariate regression analysis, lower ADAMTS13 activity and higher VWF level was related to white matter hyperintensity (WMH) [*β* = −7.31; 95% confidence interval (CI) (−9.40, −4.93); *p*<0.01; *β* = 0.17; 95% confidence interval (0.11, 0.23); *p*<0.01], subcortical infarction (SI) [(*β* = −9.22; 95% CI (−11.37, −7.06); *p*<0.01); *β* = 0.21; 95% confidence interval (0.15, 0.27); *p*<0.01] independently, but not cerebral microbleed (CMB) [(β = −2.3; 95% CI (−4.95, 0.05); *p* = 0.22); *β* = 0.02; 95% confidence interval (−0.05, 0.08); *p* = 0.63]. Furthermore, ADAMTS13 activity was independently negatively correlated with the overall CSVD burden (odd ratio = 21.33; 95% CI (17.46, 54.60); *p* < 0.01) after adjustment for age, history of hypertension, and current smoking.

**Conclusions**: Reducing ADAMTS13 activity change is related to white matter hyperintensity, subcortical infarction, but not with cerebral microhemorrhage. In addition, ADAMTS13 may have played an essential role in the progression of CSVD.

## Introduction

Cerebral small vessel disease (CSVD) is a syndrome with various structural or functional lesions involving perforating vessels that leads to parenchymal injury, which causes clinical, cognitive, neuroimaging, and neuropathological manifestation. Neuroimaging features are diverse so that the total CSVD score was created to capture the overall effect of CSVD on the brain, which incorporates white matter hyperintensity (WMH), cerebral microbleeds (CMB), lacunes, and enlarged perivascular spaces (EPVS; Huijts et al., 2013; Staals et al., 2014). The pathogenesis of the CSVD is not well known, but more evidence suggests that endothelial dysfunction is a crucial link leading to changes in cerebrovascular structure and function in patients with CSVD (Wardlaw et al., 2013; Jackman and Iadecola, 2015). Therefore, searching for endothelial biological markers will provide an essential theoretical basis for in-depth exploration of the pathogenesis of CSVD and the search for new therapeutic targets.

For CSVD, the related markers such as endothelial cell injury, inflammatory response, and coagulation/coagulation markers are well studied, and the connection between homocysteine, asymmetric dimethylarginine (ADMA), von Willebrand factor (VWF), and CSVD has been widely recognized (Wang et al., 2016; Janes et al., 2019; Nam et al., 2019). VWF is considered as a marker of endothelial dysfunction, which was synthesized in endothelial cells and released into the blood circulation *via* a constitutive pathway (released immediately after completion of molecular synthesis) or a stimulatory regulatory pathway that mediates initial platelet adhesion at sites of vascular injury (Ruggeri, 1999; Lenting et al., 2010). A disintegrin and metalloproteinase with a thrombospondin motif repeat 13 (ADAMTS13) regulate the activity of VWF by cutting ultra-long VWF multimers into smaller, less active molecules and exert its anti-inflammatory and antithrombotic properties (Gerritsen et al., 2001).

Several studies have reported that VWF levels are associated with the degree of WMH and the number of silent subcortical infarctions (Kario et al., 2001; Gottesman et al., 2009), but little is known about the association of VWF and ADAMTS13 activity with subtypes of CSVD and the overall CSVD burden. Therefore, we performed this study to investigate the independence or interactions correlation between multiple CSVD subtypes and the severity of CSVD.

## Materials and Methods

### Study Population

**Figure 1:**
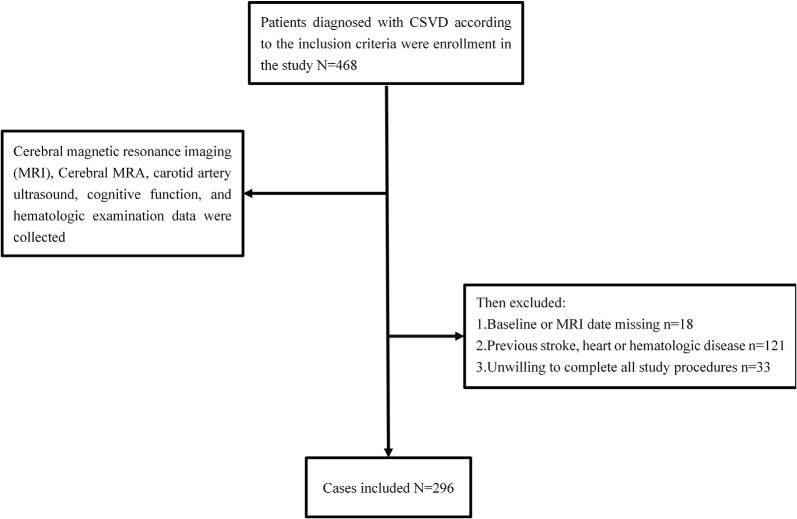
Flowchart of patients included in this study.

“Investigation on the Status of Cerebrovascular Diseases and Establishing Cohort in Shang Hai Aging Population (ISCDECSHAP)” is a prospective, population-based, and cohort study of stroke incidence and risk factors in an ageing population from Shang Hai City. The ISCDECSHAP study aimed to establish a Chinese CSVD community cohort and was approved by the ethics committee of Hua Shan Hospital and the Fifth People’s Hospital of Shanghai. Written informed consent was obtained from all the patients or their representatives before data collection (Yang et al., 2019). Based on protocol, all the subjects, at least 60 years old, performed cerebral magnetic resonance imaging (MRI), cerebral MRA, carotid artery ultrasound, cognitive function, and hematologic examination. Demographic and clinical data, including gender, age, hypertension, diabetes, smoking history, drinking history, and other health conditions were collected by neurologists through a standardized questionnaire. Three milliliters of fasting venous blood was drawn from the study subjects in the morning, all of which was completed within 1 week. According to the purpose of this study, our inclusion criteria: ≥60 years old CSVD patients; no neurological symptoms and signs; previous experience of non-specific neurological symptoms such as dizziness, vertigo, headache, and tinnitus can be included (the above symptoms should be completely relieved when evaluating the inclusion). Patients who met any one of the following criteria were excluded: history of stroke, cognitive dysfunction, heart disease, malignancies, hepatic or renal diseases, autoimmune diseases, or infection at enrollment. Contents are shown in Figure 1.

### Brain MRI and Total CSVD Score

Brain MRI imaging sequences include T1-weighted images (T1WI), T2-weighted images (T2WI), fluid-attenuated inversion recovery (FLAIR), diffusion-weighted imaging (DWI), and susceptibility-weighted imaging (SWI). The field of view (FOV) is 240 mm × 240 mm, and the matrix = 320 × 256. Coronal and transaxial views of T1WI and T2WI, and transaxial views of T2-FLAIR, DWI, and SWI were collected. The imaging diagnosis of CSVD strictly follows the international CSVD guidelines (Wardlaw et al., 2013). Subcortical lesions (SI): round or oval lesions, 3–15 mm in diameter, with cerebrospinal fluid signal intensity on T1 and T2 sequences, isointensity on DWI, hypointensity on FLAIR, and ring hyperintensity in the periphery. Perivascular space enlargement (EPVS): round or oval, and usually ≤2 mm in diameter, most pronounced in the basal ganglia, with the cerebrospinal fluid signal on T1 and T2 sequences, isointensity on DWI, and hypointensity on FLAIR. White matter hyperintensity (WMH): abnormal signals of varying sizes in periventricular and deep white matter regions, which are hyperintense on T2, FLAIR, and isointense or hypointense on T1. Cerebral microbleed (CMB): it is round on SWI sequence, less than 10 mm in diameter, and hypointense. WMH was graded using a semi-quantitative Fazekas scale (Hainsworth et al., 2015), and high WMH was defined if the scale scored 2–3. According to Staals et al. (2014), the overall burden score of CSVD was rewarded with 1 point whenever one of the following occurred: ≥1 SI (<15 mm); ≥1 CMBs (<5 mm); moderate-severe basal ganglia PVS (semiquantitative scale 2–4 grades; Maclullich et al., 2004); deep WMH (Fazekas score 2/3) or periventricular WMH (Fazekas score 3). The total score of CSVD is 0–4 points, a higher score indicating that the CSVD is more severe. The MRI was independently evaluated by two neurologists, and inter-observer agreement values for the presence of CMB, WMH, EPVS, and lacune were 0.80, 0.83, 0.78, and 0.79, respectively. Any disagreement regarding the presence of CSVD features was resolved by consensus with the third neuroimaging expert.

### Measurement of ADAMTS13 Activity and VWF Level

Human VWF ELISA kit (Abcam) was used to quantitatively measure the VWF level directly in human fasting venous plasma samples obtained on admission. The samples were centrifuged (2,000 *g* for 5 min) immediately after collection, and plasma samples were stored in a −80 refrigerator. The assay was a sandwich ELISA and measurements were conducted according to the manufacturer’s guidelines. ADAMTS13 enzyme activity was measured by the fluorescent substrate method, AnaSpecUSA provided FRETS-VWF73 (ADAMTS13 fluorescent substrate), and the percentage of enzyme activity indicated that the standard plasma activity was set at 100. The operation steps were performed according to the assay (Kokame et al., 2005). Standard plasma is a mixture of plasma from 20 healthy subjects and provided by the Physical Examination Center of Shanghai Fifth People’s Hospital.

### Statistical Analysis

SPSS 21.0 statistical software was applied for data analysis. For each demographic and clinical feature, normal distribution continuous variables were presented as mean ± standard deviation and compared by an independent sample *t*-test. Abnormal distribution continuous variables were presented as median (interquartile range) and compared by a nonparametric test. The categorical variables are expressed in frequency (percentage) and are expressed in χ^2^ test or Fisher exact test. Associations between circulating biomarker (ADAMTS13 and VWF) levels and neuroimaging markers of CSVD and overall CSVD burden was performed by regression analysis. Multivariable linear regression was then conducted for each marker of CSVD in separate models adjusting for age, sex, hypertension, diabetes, smoking history, drinking history, and all brain variables. Kendall’s tau-b correlation analysis was used to evaluate the correlation between the ADAMTS13 activity and the total CSVD score. For logistic regression models, age, sex, and variables showing a *p* < 0.1 on the respective univariate analyses were included in models. A *p*-value of 0.05 was considered significant.

## Results

### Clinical Characteristics of Study Patients

A total of 296 patients with CSVD were included in this study, of which 125 were male. The mean age of the sample was s 69.0 years (SD 7.0), the median VWF level was 1.28 (1.01–1.50) IU/ml, and the ADAMTS13 activity was 88.04% (SD10.57). SI accounted for 42.3% (*n* = 125) of the total sample, CMB for 28.0% (*n* = 83), and high WMH (Fazekas score of 2–3) for 41.6% (*n* = 123). SI has higher total cholesterol than no SI (*p* < 0.01). We found that the high WMH and SI group had significantly increased VWF levels (*p* < 0.01) and decreased ADAMTS13 activity (*p* < 0.01) compared with the control group, but there was no significant difference in the changes of the above parameters in the CMB group (Table 1).

**Table 1:** Sample characteristics by brain variable.

	Total Sample	No SI	SI		Low WMH	High WMH		No CMB	CMB	
	*n* = 296	*n* = 171	*n* = 125	*P*	*n* = 162	*n* = 134	*P*	*n* = 213	*n* = 83	*P*
Age, years	69 ± 7.0	66 ± 5.5	72 ± 7.7	<0.001	66 ± 6.3	72 ± 7.1	0.010	68 ± 7.2	70 ± 7.3	0.066
Male, *n* (%)	125 (42.2)	80 (46.8)	62 (49.6)	<0.632	75 (46.3)	67 (50.0)	0.526	105 (49.3)	37 (44.6)	0.466
BMI, kg/m2	24.7 ± 4.0	24.5 ± 4.0	24.6 ± 3.1	<0.762	24.7 ± 3.7	24.4 ± 3.5	0.243	24.6 ± 3.6	24.4 ± 3.7	0.592
Alcohol use, *n* (%)	72 (24.3)	27 (15.8)	45 (36)	<0.001	26 (16.0)	46 (34.3)	<0.001	42 (19.7)	30 (36.1)	<0.001
Current smoking, *n* (%)	99 (33.5)	29 (17.0)	70 (56.0)	<0.001	35 (21.6)	64 (47.8)	<0.001	72 (33.8)	27 (32.5)	0.835
Hypertension, *n* (%)	152 (51.4)	63 (36.8)	89 (71.2)	<0.001	54 (33.3)	98 (73.1)	<0.001	93 (43.7)	59 (71.1)	<0.001
Diabetes Mellitus, n (%)	80 (27.0)	30 (17.4)	52 (40.0)	<0.001	35 (21.6)	45 (36.2)	0.834	47 (22.1)	33 (39.8)	0.002
Total cholesterol, mmol/L	4.40 ± 1.20	4.07 ± 1.20	4.85 ± 1.04	<0.007	4.39 ± 1.16	4.41 ± 1.23	0.222	4.37 ± 1.21	4.47 ± 1.14	0.545
HDL cholesterol, mmol/L	1.35 ± 0.59	1.38 ± 0.68	1.29 ± 0.44	<0.212	1.29 ± 0.57	1.42 ± 0.62	0.062	1.38 ± 0.59	1.27 ± 0.60	0.153
LDL cholesterol, mmol/L	3.33 ± 0.79	3.30 ± 0.82	3.32 ± 0.77	<0.748	3.37 ± 0,79	3.25 ± 0.79	0.180	3.26 ± 0.76	3.46 ± 0.85	0.048
VWF:Ag, IU/ml	1.31 ± 0.28	1.23 ± 0.31	1.41 ± 0.18	<0.001	1.25 ± 0.26	1.38 ± 0.27	<0.001	1.31 ± 0.28	1.29 ± 0.27	0.462
ADAMTS13 activity (%)	88.01 ± 10.57	91.44 ± 10.47	83.28 ± 8.61	<0.006	90.29 ± 11.40	85.24 ± 8.60	0.001	88.37 ± 10.81	87.10 ± 9.74	0.545

*Note: CMB, Cerebral Microbleed; IQR, Interquartile range; SI, Subcortical infarct; WMH, Median White Matter Hyperintensity; LDL cholesterol, low-density lipoprotein cholesterol; HDL cholesterol, high-density lipoprotein cholesterol*.

### Associations Between CSVD and the ADAMTS13 Activity

The association between different CSVD neuroimaging markers and ADAMTS13 activity was investigated. Adjusted for age, sex, alcohol, smoking, hypertension, and diabetes, lower ADAMTS13 activity and higher VWF level were related with high WMH [β= −6.26; 95% CI (−7.55, −2.82) *p*<0.01; *β* = 0.18; 95% CI (0.12, 0.25); *p*<0.01] and SI [*β* = −5.19; 95% CI (−7.55, −2.82) *p*<0.01; *β* = 0.13; 95% CI (0.07, 0.20); *p*<0.01]. Model II includes all brain variables, The presence of SI and WMH still remain associated with lower ADAMTS13 activity and higher VWF level (Table 2; Supplementary Table 1). In addition, we calculated the ADAMTS13:VWF ratio; the results showed that compared with ADAMTS13 alone, ADAMTS13:VWF ratio had a more pronounced association with SI [*β* = −23.24; 95% CI (−29.31, −17.16); *p*<0.01] and WMH [*β* = −17.35; 95% CI (−24.35, −10.35); *p* < 0.01] (Table 3). The final ADAMTS13:VWF model explained 32.3% (adjusted R squared) of the variance, which was higher than the 28.2% (adjusted R squared) of the ADAMTS13-only model in this study.

**Table 2:** Multivariate analysis of ADAMTS13 levels with brain variable.

	ADAMTS13 (Mode I)		ADAMTS13 (Mode II)	
	Coefficient	95% confidence interval	Coefficient	95% confidence interval
Subcortical infarct	−6.26*	[−9.08, −3.45]	−9.22*	[−11.37, −7.06]
Cerebral microbleeds	−0.51	[−3.17, 2.15]	−2.30	[−4.95, 0.05]
WMH	−5.19*	[−7.55, −2.82]	−7.31*	[−9.40, −4.93]

Note: *p < 0.01. Mode I, adjusted for age, sex, alcohol use, current smoking, hypertension, diabetes mellitus; Mode II, Adjusted Mode I with all brain variables *R^2^* = 0.28.

**Table 3:** Multivariate analysis of ADAMTS13:VWF ratio with brain variable.

	ADAMTS13:VWF ratio (Mode I)		ADAMTS13:VWF ratio (Mode II)	
	Coefficient	95% confidence interval	Coefficient	95% confidence interval
Subcortical infarct	−14.89*	[−20.76, −9.01]	−23.24*	[−29.31, −17.16]
Cerebral microbleeds	4.25	[−1.22, 9.23]	−3.30	[−9.20, 2.38]
WMH	−12.22*	[−17.23, −7.26]	−17.35*	[−24.35, −10.35]

Note: *p < 0.01. Mode I, adjusted for age, sex, alcohol use, current smoking, hypertension, diabetes mellitus; Mode II, adjusted model with all brain variables *R^2^* = 0.32.

### Associations Between Total CSVD Burden and the ADAMTS13 Activity

Patients were classified into four groups by ADAMTS13 activity and VWF level quartiles, respectively. In our study population, approximately half of the CSVD total score of patients was 1 (26.3%) or 2 (32.1%). A number of patients scored 3 (28.7%) and 4 (16.2%). Of the patients with a CSVD score of 4, the majority had ADAMTS13 activity (78.8%) among ADAMTS13_Q1<80.3%_. In addition, the correlation analysis shows that the ADAMTS13 activity was negatively related to the total CSVD score (Kendall’s tau-*b* = −0.48, *p*<0.01; Table 4). In the adjusted statistical model (adjusted for age, sex, alcohol use, current smoking, hypertension, and diabetes mellitus), the increase in total CSVD score was negatively correlated with ADAMTS13_Q1<80.3%_ (*OR* = 21.33, 95% CI (17.46, 54.60) *p* < 0.01) and ADAMTS13_Q280.3–89.0%_ (*OR* = 2.89, 95% CI (1.75, 17.50) *p* < 0.05), respectively. Moreover, lower VWF levels (VWF_Q1<1.14 IU/ml_) can be regarded as a protective factor for CSVD (*OR* = 0.37, 95% CI (0.20, 0.66) *p* < 0.01; Table 5).

**Table 4:** The characteristics of ADAMTS13 activity of the patients with total CSVD score.

	Total CSVD Score				
	*n* = 296	1 (*n* = 78)	2 (*n* = 95)	3 (*n* = 85)	4 (*n* = 38)
**ADAMTS13 activity, *n* (%)**			
Q1_<80.3%_		2 (2.6)	10 (10.5)	29 (34.1)	30 (78.8)
Q2_80.3−89.0%_		18 (37.5)	24 (25.3)	27 (31.8)	4 (10.5)
Q3_89.0−97.1%_		25 (32.0)	28 (29.5)	20 (23.5)	4 (10.5)
Q4_>97.1%_		33 (42.3)	33 (34.7)	9 (10.6)	0

*Note: Kendall’s tau-*b* = 0.46, *p* < 0.01*.

**Table 5:** Multiple ordinal regression analysis of total CSVD score with ADAMTS13.

	OR	95% confidence interval	*p*-value
**VWF:Ag (IU/ml)**			
Q1_<1.14 IU/ml_	0.37	[0.20, 0.66]	<0.01
Q2_1.33−1.14 IU/ml_	0.68	[0.38, 1.23]	<0.21
Q3_1.33−1.51 IU/ml_	0.50	[0.30, 0.79]	<0.05
Q4_ >1.51 IU/ml_		Reference	
**ADAMTS13 activity (%)**			
Q1_<80.3%_	21.33	[17.46, 54.60]	<0.01
Q2_80.3−89.0%_	2.89	[1.75, 17.50]	<0.01
Q3_89.0−97.1%_	1.70	[0.91, 3.16]	<0.09
Q4_>97.1%_		Reference	

*Note: Adjusted for age, sex, alcohol use, current smoking, hypertension, diabetes mellitus*.

## Discussion

In our study, we investigated the association between plasma VWF level, ADAMTS13 activity, and neuroimaging features of CSVD, including the overall CSVD burden. After adjustment for established CSVD-related risk factors, we confirmed that ADAMTS13 activity was reversely correlated to WMH, SI, and overall CSVD burden independently.

The prevalence of CSVD in the elderly population over 60 years can reach 60%. Studies have shown that about 45% of dementia and 20% of stroke is caused by CSVD (Sudlow and Warlow, 1997; Gorelick et al., 2011). Furthermore, CSVD may double the risk of recurrent stroke (Debette and Markus, 2010). In recent years, large population-based studies report that low ADAMTS13 activity was correlated with a risk of ischemic stroke (Sonneveld et al., 2015) and dementia (Wolters et al., 2018) independent of other known demographic and cerebrovascular risk factors, but studies on the relationship between ADAMTS13 activity and CSVD are little. The association between VWF and neuroimaging markers of CSVD has been demonstrated, including silent lacunar cerebral infarcts, periventricular hyperintensity, and deep white matter hyperintensity (Kario et al., 2001; Gottesman et al., 2009; Nagai et al., 2012). However, it has not reflected changes between VWF with the overall CSVD burden. In our study, we demonstrated that higher VWF level and lower ADAMTS13 activity correlated with WMH and SI, but not with CMB after adjusting for traditional risk factors and brain variables. The complex mechanism involved is unknown, but there are several possible explanations as follows.

First, low ADAMTS13 activity may be associated with endothelial cell dysfunction. Endothelial dysfunction may lead to increased permeability of the blood-brain barrier (BBB), which not only interrupts the oxygen and nutrient supply to the brain but also leads to extravasation of blood components that damage the surrounding white matter regions (Zlokovic, 2008; Gorelick et al., 2011; Xu et al., 2015). Cao et al. (2019) observed that ADAMTS13-deficient mice promote BBB disruption and reduce microvascular and capillary perfusion, and cerebral blood flow by changing endothelial junctions. However, there is a tight link between WMH and BBB impairment that plays a crucial role in early white matter degeneration (Kerkhofs et al., 2021). For SI, its underlying mechanism may have partially overlapping pathophysiology with WMH, but somewhat different (Wardlaw et al., 2015; Lin et al., 2017). VWF, an indicator of endothelial dysfunction, is the first step in mediated adhesion of platelets to damaged endothelial cells in thrombus formation. When endothelial cells are dysfunctional, VWF will be released in the form of large-molecular-weight multimers, which aggravate vascular damage (Lip and Blann, 1997). ADAMTS13 alleviates thrombogenesis and inflammation by adjusting VWF size and regulates microvascular thrombosis by altering the interaction of VWF with platelets (Pillai et al., 2016; Gogia et al., 2017). A study has reported that increased levels of functional VWF accelerate the formation of more microthrombi in multiple SI elderly patients (Kario et al., 2001). In this study, we obtained similar results in VWF and found that low ADAMTS13 activity was an independent risk factor for multiple SIs, and there was no association between ADAMTS13 activity and VWF levels, similar to previous studies (Andersson et al., 2012; Sonneveld et al., 2015; Denorme et al., 2017).The possible reason is that low local ADAMTS13 activity is not sufficient to cleave the supramaximal VWF multimers, eventually leading to the formation of microthrombiin microvascular injury. A second possible reason is that ADAMTS13 activity accelerates atherosclerosis. In animal models, lack of ADAMTS13 was found to promote the formation of plaques and vascular inflammation by generating signals for recruitment and extravasation of monocytes in the early stages of atherosclerosis, which are the pathological changes associated with CSVD (Gandhi et al., 2012; Jin et al., 2012). Ultimately, this may trigger or worsen the development of CSVD.

Furthermore, we confirmed that the severity of the overall CSVD burden was negatively correlated with ADAMTS13 activity. In another study, Nezu et al. (2015) observed that endothelial dysfunction was related to the severity of CSVD by using flow-mediated dilation (FMD) to measure endothelium-dependent vasodilation. However, ADAMTS13 was shown to be able to augment microvascular endothelial endothelium-dependent dilation (EDR) dependent on PI3K/Akt pathway *in vivo* (Zhou et al., 2019). Therefore, this implies ADAMTS13 may have played an essential role in the development of CSVD.

The innovation of this study is that we first reported the association between plasma VWF level, ADAMTS13 activity, and neuroimaging features of CSVD, including the overall CSVD burden in the community cohort. However, there are some limitations as follows. First, this study is a single-center cross-sectional study, with a relatively small number of study subjects and the included biological marker indicators are not comprehensive enough. Because we could not integrate multiple CSVD biomarkers into a panel, it will help us understand the pathogenesis of CSVD better. Second, the activity of ADAMTS13 was also demographically statistically different among different races and regions, and the subjects we included were all from Shanghai, China, so our results need to be validated in a more extensive prospective study.

In conclusion, reducing ADAMTS13 activity is related to white matter hyperintensity and subcortical infarction, but not with cerebral microhemorrhage. In addition, this implied that ADAMTS13 may have played an essential role in the development of CSVD. Exploring the differences of various biomarkers between various neuroimaging features of CSVD, and the association of these markers with clinical manifestations of CSVD will help to deeply elucidate the pathogenesis of CSVD and may provide a new theoretical basis and new targets for the early prevention and treatment of CSVD.

## Data Availability Statement

The raw data supporting the conclusions of this article will be made available by the authors, without undue reservation.

## Ethics Statement

The studies involving human participants were reviewed and approved by Shanghai Fifth People’s Hospital, Fudan University. The patients/participants provided their written informed consent to participate in this study.

## Author Contributions

WS: conceptualization, data collection, experiment, formal analysis, and writing original draft. SZ and YL: data collection. WL: validation, supervision, and experiment. XY and DW: review and editing, provide guidance. All authors contributed to the article and approved the submitted version.

## Conflict of Interest

The authors declare that the research was conducted in the absence of any commercial or financial relationships that could be construed as a potential conflict of interest.

## Publisher’s Note

All claims expressed in this article are solely those of the authors and do not necessarily represent those of their affiliated organizations, or those of the publisher, the editors and the reviewers. Any product that may be evaluated in this article, or claim that may be made by its manufacturer, is not guaranteed or endorsed by the publisher.
