# Supplementary material for: The Relationship Between ADAMTS13 Activity and Overall Cerebral Small Vessel Disease Burden: A Cross-Sectional Study Based on CSVD
Source: Front Aging Neurosci. 2021 Oct 8;13:738359. doi: 10.3389/fnagi.2021.738359 (PMC8531192; doi:10.3389/fnagi.2021.738359)
Supplement: Supplementary file 1 [file Data_Sheet_1.docx]

Supplement table1.Multivariate analysis of VWF levels with Brain Variable

|  | VWF (Mode I) |  | VWF (Mode II) |
| --- | --- | --- | --- |
|  | Coefficient 95% confidence interval |  | Coefficient 95% confidence interval |
| Subcortical infarct | 0.18* [0.12,0.25] |  | 0.21* [0.15, 0.27] |
| Cerebral microbleeds | -0.02 [-0.09,0.06] |  | 0.02 [-0.05,0.08] |
| WMH | 0.13* [0.07,0.20] |  | 0.17* [0.11,0.23] |

Note: *p＜0.01; Mode I: adjusted for age, sex, alcohol use, current smoking, hypertension, diabetes mellitus; Mode II: Adjusted Model With All Brain Variables R2=0.21
